# Labeled extracellular vesicles can be found in the blood plasma shortly after intrauterine infusion in bovine

**DOI:** 10.1590/1984-3143-AR2024-0064

**Published:** 2024-08-26

**Authors:** Mariani Farias Fiorenza, Alessandra Bridi, Gislaine dos Santos, Paola Maria Rosa, Luana Alves, Juliana Germano Ferst, Priscila Assis Ferraz, Guilherme Pugliesi, Ky Pohler, Felipe Perecin, Flávio Vieira Meirelles, Juliano Coelho da Silveira

**Affiliations:** 1 Departamento de Medicina Veterinária, Faculdade de Zootecnia e Engenharia de Alimentos, Universidade de São Paulo, Pirassununga, SP, Brasil; 2 Departamento de Reprodução Animal, Faculdade de Medicina Veterinária e Zootecnia, Universidade de São Paulo, Pirassununga, SP, Brasil; 3 Department of Animal Science, Texas A&M University, College Station, Texas, United States

**Keywords:** nanoparticles, migration, uterus, bovine, neutrophils

## Abstract

This study explored the migration of follicular fluid (FF)-derived extracellular vesicles (EVs) of the uterine environment to the bloodstream and their interaction with neutrophils *in vivo* and *in vitro*. For the *in vivo* experiment, six Nellore heifers (*Bos indicus*) received an intrauterine infusion seven days after ovulation with 1X PBS only (sham group; n=1), 1X PBS stained with lipophilic dye PKH26 (control group; n=2), or FF-derived EVs stained with PKH26 (treated group; n=3). Plasma was collected at 0, 10, 30, 60-, 180-, 360-, 720-, and 1440-min post-infusion to obtained EVs for analysis by nano flow cytometry. Labeled EVs were present in the bloodstream at 30- and 60-min post-infusion in the treatment group. Additionally, plasma derived-EVs from all groups were positive for Calcein-AM, Alix, Syntenin, and Calnexin, which confirm the presence of EVs. The second experiment utilized the plasma-derived EVs from the heifers from 30 and 60 min timepoints to evaluate if neutrophils can uptake EVs *in vitro*. As results, it was possible to observe the presence of labeled EVs in neutrophils treated with plasma derived-EVs from the treatment group. In summary, our results suggest that labeled EVs can migrate from the uterine environment rapidly and interact with circulating immune cells in bovine.

## Introduction

Extracellular vesicles (EVs) are involved in reproductive processes as mediators of intercellular communication (De Ávila et al., 2019). EVs are cargo-bearing nanoparticles released by cells into the extracellular space (Couch et al., 2021). Once shed from the parent cell, EVs can migrate and deliver their cargo to recipient cells, eliciting biological responses (Crawford, 1971). EVs modulates processes, such as embryo development and immunomodulation (De Ávila et al., 2019) in important periods as maternal recognition of pregnancy (Bridi et al., 2021). In ruminants, classical definition describes that maternal recognition of pregnancy stars around day 15; however, modulation of uterine transcriptome can start as early as day 7 in the presence of an embryo (Sponchiado et al., 2017). As the pregnancy develops, this communication between mother and embryo passes from being a local event to be a systemic modulation in the maternal body (Oliveira et al., 2008). However, more studies are necessary to better understand the real contribution of an early modulation of pregnancy in cows. Notably, EVs may enhance embryo quality (Leal et al., 2022), cellular proliferation and embryonic maternal communication (Kurian and Modi, 2019; Bridi et al., 2021). Cows exhibit EVs within their plasma throughout various stages of their reproductive cycle, exhibiting phenotypic variations (Pohler et al., 2017).

However, most of the interactions are primarily described as local effects. There remains a gap in understanding whether EVs have the capacity to exit the uterus and interact with circulating immune cells in the maternal body. The hypothesis of the experiment is that labeled EVs can leave the uterine environment and interact with immune cells in the bloodstream of cows in the early days of the estrus cycle. Thus, the objective of this study is to investigate the ability of labeled EVs to migrate from the uterine environment to the systemic circulation and interact with immune cells on day 7 post-ovulation.

## Methods

### Experimental design

This study was divided into two experiments: first, we performed an *in vivo* experiment to analyze the exit of EVs from the uterus to the bloodstream. Nellore heifers (*Bos indicus*) with pre-ovulatory follicles received 2.5 mL of a synthetic GnRH analog (Ouro Fino, Sincrogest). Only the animals that ovulated 2 days after the GnRH injection were included in the experiment. Seven days post-ovulation, six heifers were divided aleatory into three groups: 1X PBS only (sham group; n=1), 1X PBS stained with lipophilic dye PKH26 (control group; n=2), follicular fluid (FF)-derived EVs stained with PKH26 (treated group; n=3). Day 7 after ovulation was chosen to approximate early pregnancy hormonal levels. Ten mL of FF was used to isolate EVs using ultracentrifugation method (De Ávila et al., 2020) for the intrauterine infusion. The EVs were stained with PKH26 (Sigma-Aldrich, MIDI26-1KT; 10 μL) according to the manufacturer instructions and loaded into a 0.25 mL straw. Epidural anesthesia was induced using 4 mL of lidocaine hydrochloride, and intrauterine infusion of EVs was performed into the ipsilateral uterine horn to the corpus luteum (CL). At 0, 30, 60, 180-, 360-, 720-, and 1440-min post-infusion, blood samples from the jugular vein were collected for isolation of EVs. Plasma derived-EVs were isolated by size exclusion chromatography (SEC; Izon qEV35) according to the manufacturer instructions and analyzed for presence of positive events/μL for PKH26 by nano flow cytometry (nFCM). Plasma derived-EVs were stained with calcein-AM (Sigma-Aldrich; 17783; 1 μM). EV immunophenotyping used Alix^-PE^ (sc-53540; 1:50), CD81^-PE^ (ab81436; 1:50), Syntenin (sc-515538; 1:50), and AF488 secondary antibody (A11001; 1:2000). Calnexin^-AF488^ (sc-376768; 1:50) detected contamination. Samples were analyzed by Cytoflex (Beckman Coulter). The second experiment was performed *in vitro*. Plasma-derived EVs obtained during the i*n vivo* experiment were isolated by size exclusion chromatography and used to treat *in vitro* neutrophils from multiparous Nellore cows, aiming to analyze the EVs uptake by these cells. Neutrophils were isolated as previously described (Fiorenza et al., 2021). The neutrophils were then treated with plasma derived-EVs collected at 30 and 60 minutes post-intrauterine infusion. After 180 min of culture, cells were harvested, fixed, stained with Hoechst 33342 DNA dye (Sigma-Aldrich; B2261) and mounted in a glass slide to be analyzed by fluorescence microscope (Leica Thunder 3D Imager DMi8), with a scale bar set to 50 µm. Hoechst was read in filter LED405 and PKH26 in Y3. The protocol was submitted to the University of São Paulo Research Ethics Committee (n˚ 1484220324). The present work was performed with the goal to describe the EVs dynamic in an *in vivo* experiment. Due to the experimental design, we did not perform any statistical analysis.

## Results

### *In vivo* experiment

#### Nano flow cytometry of EVs from plasma

**Figure 1:**
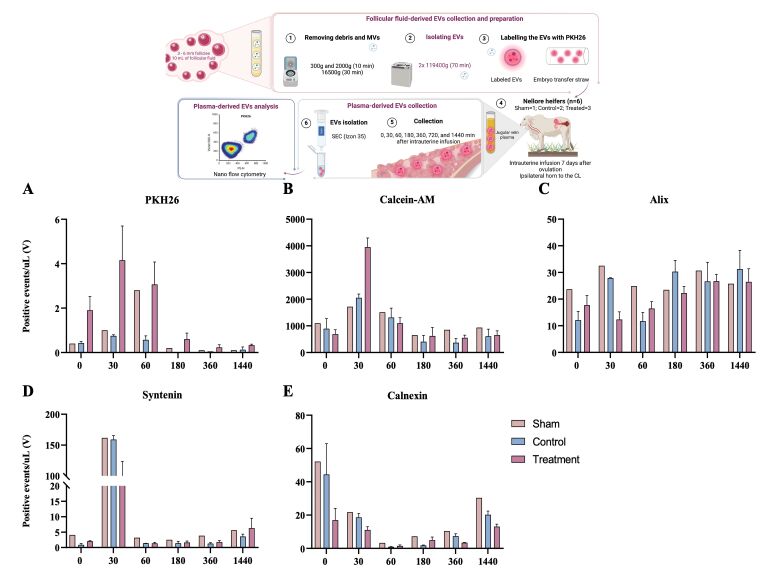
Nano flow cytometry analysis of plasma-derived extracellular vesicles. (A) nFCM analysis revealed the presence of PKH26-labeled EVs in plasma samples at 30- and 60-minutes post intrauterine infusion with EVs derived from FF, with a gradual decrease observed over time until 1440 minutes. (B) Positive events/uL for calcein-AM was present in all samples over time. (C, D, and E) All plasma derived-EVs isolated from the different groups had positive events for Alix, Syntenin and Calnexin.

To observe the capacity of EVs infused in the uterus to arrive at the bloodstream, nFCM analysis was performed. The results demonstrated the presence of 1 positive event/µL in sham (Figure 1A). In the control group, we observed 0.75±0.05 and 0.57±0.17 positive events/ µL in 30 and 60 min, respectively (Figure 1A). In the treated group, it was possible to observe 4.15±1.53 and 3.65±1.01 positive events/µL for PKH26 after 30 and 60 min after intrauterine infusion, respectively. Additionally, we observed a lower number of PKH26 labeled EVs from 60 min until 1440 min (Figure 1A) in the treated group (Figure 1A). Positive events for Calcein-AM (Figure 1B), Alix (Figure 1C), Syntenin (Figure 1D) and Calnexin (Figure 1E) in all groups at different timepoints.

### *In vitro* experiment

#### Extracellular vesicles from the *in vivo* experiment can be uptake by neutrophils *in vitro*

**Figure 2:**
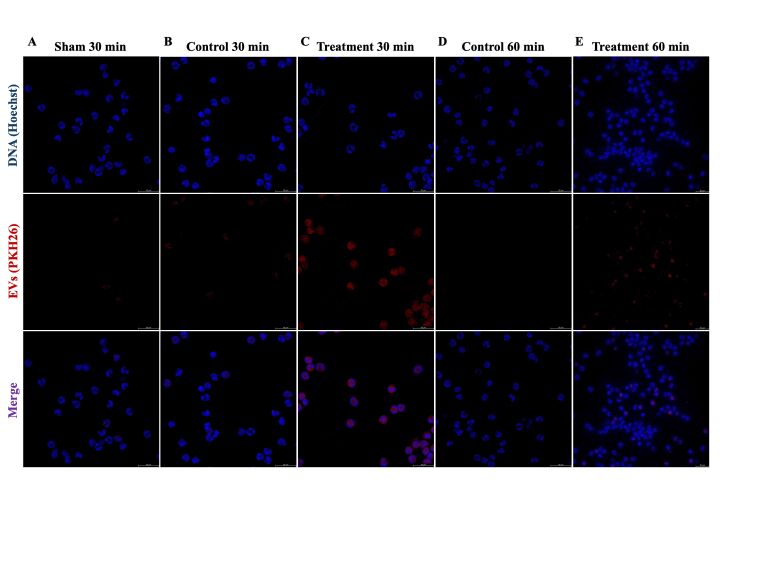
Neutrophils treated with plasma-derived EVs from 30 to 60 min after intrauterine infusion. (A) Neutrophils treated with plasma derived-EVs collected 30 min after intrauterine infusion from the sham group, respectively. (B and C) Neutrophils treated with plasma derived-EVs collected 30 min after intrauterine infusion from the control and treated groups, respectively. (D and E) Neutrophils treated with plasma derived-EVs collected 60 min after intrauterine infusion from control and treated groups. Images were obtained using 10x magnification.

After observing the presence of PKH26-labeled EVs in plasma following intrauterine infusion, our investigation delved into the potential interaction between these EVs and neutrophils. For this, neutrophils were treated with plasma-derived EVs from the *in vivo* experiment collected 30 and 60 min after intrauterine infusion due to their higher count of positive events/ µL in nFCM. It was possible to observe the presence of PKH26 labeled EVs in neutrophils treated with EVs derived from the treatment group 30 (Figure 2C) and 60 (Figure 2E), the same presence was not observed when neutrophils were treated with EVs derived from the sham (Figure 2A) and control group (Figure 2B-D).

## Discussion

This study demonstrates, for the first time, the movement of EVs from the uterine environment to the bloodstream in bovine model. nFCM and immunofluorescence were used to detect PKH26-labeled EVs in plasma samples at various time points after intrauterine infusion. Furthermore, EVs isolated from plasma samples from cows that received intrauterine infusion of PKH26-labeled EVs were used to treat neutrophils, indicating their uptake by these cells. Despite limitations such as small animal numbers per group, PKH26 labeling efficiency, and low detection events in nFCM, it is notable that the sample volume used for plasma derived-EVs isolation represented only 0.0033% of total blood volume from a cow (Möllerberg et al., 1975). All treated animals showed increased PKH26-positive events at 30- and 60-minutes post-infusion. Additionally, plasm derived-EVs were positive for markers including Alix, Syntenin, Calcein-AM, and Calnexin, which confirm the presence of EVs in the isolate of all groups according to MISEV guidelines (Welsh et al., 2024).

Previous studies in mice demonstrated that administration for EVs can reach different tissues all over the body and have a fast clearance by immune cells, presenting a half-life of minutes and being removed after 30 to 60 min after intravenous administration (Takahashi et al., 2013; Lai et al., 2014), corroborating with the results found in the present study. These findings are important to pave the role of EVs in the embryo-maternal communication outside the uterine environment and may contribute to the immune response during early pregnancy development.

In conclusion, the *in vivo* model experiment demonstrates that PKH26 labeled EVs can leave the uterine environment and may be detected in the bloodstream. Moreover, they interact with neutrophils, as demonstrated in the *in vitro* model.
